# Validation of reference genes for normalization of gene expression by qRT-PCR in a resveratrol-producing entophytic fungus (*Alternaria* sp. MG1)

**DOI:** 10.1186/s13568-016-0283-z

**Published:** 2016-11-08

**Authors:** Jin-xin Che, Jun-ling Shi, Yao Lu, Yan-lin Liu

**Affiliations:** 1 College of Food Science and Engineering, Northwest A & F University, 28 Xinong Road, Yangling, 712100 Shaanxi People’s Republic of China; 2 Key Laboratory for Space Biosciences and Space Biotechnology, School of Life Sciences, Northwestern Polytechnical University, Xi’an, 710072 Shaanxi People’s Republic of China

**Keywords:** qRT-PCR, Reference genes, α-Tubulin, Elongation factor 1, *Alternaria* sp.

## Abstract

*Alternaria* sp. MG1, an endophytic fungus isolated from *Vitis vinifera*, can independently produce resveratrol, indicating that this species contains the key genes for resveratrol biosynthesis. Identification of these key genes is essential to understand the resveratrol biosynthesis pathway in this strain, which is currently unknown in microorganisms. qRT-PCR is an efficient and widely used method to identify the key genes related to unknown pathways at the level of gene expression. Verification of stable reference genes in this strain is essential for qRT-PCR data normalization, although results have been reported for other *Alternaria* sp. strains. In this study, nine candidate reference genes including *TUBA*, *EF1*, *EF2*, *UBC*, *UFD*, *RPS5*, *RPS24*, *ACTB* and *18S* were evaluated for expression stability in a diverse set of six samples representing different growth periods. We compared cell culture conditions and an optimized condition for resveratrol production. The comparison of the results was performed using four statistical softwares. A combination of *TUBA* and *EF1* was found to be suitable for normalization of *Alternaria* sp. MG1 in different developmental stages, and *18S* was found to be the least stable. The reference genes verified in this study will facilitate further research to explore gene expression and molecular mechanisms as well as the improvement of secondary metabolite yields in *Alternaria* sp. MG1. To our knowledge, this is the first validation of reference genes in *Alternaria* with the capability to produce resveratrol. Additionally, these results provide useful guidelines for the selection of reference genes in other *Alternaria* species.

**Electronic supplementary material:**

The online version of this article (doi:10.1186/s13568-016-0283-z) contains supplementary material, which is available to authorized users.

## Introduction

Many important bioactive compounds are widely used in medical services and health care (Khan 2016; Larsen and Matchkov 2016; Morata et al. 2015). Many of these compounds are either microbial metabolites or their semi-synthetic derivatives (Golinska et al. 2015; Sessitsch et al. 2013; Stepniewska and Kuzniar 2013). In the microbial population, endophytes are a large group which may contain millions of different species, but only a minority of them have been studied (Sessitsch et al. 2013). Several endophytic fungi (*Alternaria* sp.) were identified previously that are capable of independent resveratrol production (Shi et al. 2012). Although fundamental physiological research has been performed (Zhang et al. 2013a, b), the metabolic pathways and cellular processes remain to be elucidated.

Gene expression profiling is an informative technique to investigate biological systems (Li et al. 2015). The method of qRT-PCR (quantitative real time PCR) can measure gene expression across different sample populations (Derveaux et al. 2010; Wong and Medrano 2005). However, there are many factors that can influence the accuracy of the results such as the quality and quantity of mRNA templates or amplification efficiency. Generally, normalizing expression of the target genes to one or several reference genes provide an efficient way to reduce these effects and increase the relevance of the results (Huggett et al. 2005; Marabita et al. 2016). However, the use of inappropriate reference genes that change expression levels under different conditions can cause interpretation errors. Thus, the choice of appropriate reference genes for normalization is a prerequisite for qRT-PCR assay.

In recent years, validation of reliable reference genes before their use for normalization has been performed for many species, such as *Talaromyces marneffei* (Dankai et al. 2015), *Staphylococcus aureus* (Sihto et al. 2014), *Beauveria bassiana* (Zhou et al. 2012), *Oenococcus oeni* (Sumby et al. 2012) and others. Commonly used reference genes for these fungi include the genes encoding the *18S* ribosomal RNA (*18S*), ubiquitin fusion degradation protein (*UFD*), ribosomal protein (*RPS*), elongation factor (*EF*), β-actin (*ACTB*), α-tubulin (*TUBA*), ubiquitin-conjugating enzyme (*UBC*), and glyceraldehyde-3-phosphate dehydrogenase (*GAPDH*) (Kozera and Rapacz 2013). The previously reported reference genes for normalizing qRT-PCR data in fungi, especially in *Alternaria* sp. are β-tubulin (*TUB*) for *A. alternata* (Baez-Flores et al. 2011; Buzina et al. 2008) and *A. brassicicola* (Sellam et al. 2006), benA for *A. alternata* (Saha et al. 2012), 18S for *A. infectoria* (Fernandes et al. 2014), and elongation factor 1 (*EF1*) for *A. brassicicola* (Cho et al. 2014). Genes that show stable expression under many conditions may differ in microorganisms due to different organization structures and when different genes are expressed. Therefore, it is necessary to identify reliable reference genes in *Alternaria* sp. MG1 for use in qRT-PCR assay.

The aim of this study was to identify the most stable reference genes in *Alternaria* sp. under different growth conditions and resveratrol production conditions. Genes that show relatively similar levels of expression under all conditions could serve as reference genes that would be appropriate for comparison in the qRT-PCR assay of genes whose expression may vary during changes in metabolism or during resveratrol biosynthesis. Several software applications were used for analysis of candidate reference genes. These programs allowed evaluation of appropriate reference genes under given experimental conditions using statistical methods, such as Bestkeeper (Pfaffl et al. 2004), geNorm (Vandesompele et al. 2002), and Normfinder (Andersen et al. 2004).

## Materials and methods

### Microorganism

*Alternaria* sp. MG1 (code: CCTCC M 2011348), a strain previously isolated from the cob of Merlot grape (Shi et al. 2012), was used in the study. It was maintained at the China Center for Type Culture Collection (Wuhan, China). For preparation of *Alternaria* sp. MG1 cells, the strain was inoculated into a 250-mL flask containing 100 mL PDB (liquid potato-dextrose broth, pot,ato 200 g with 20 g glucose in 1000 mL tap-water). The cultivation was carried out at 28 °C and 120 rpm in a rotary shaker. According to the growth curve analysis (Additional file 1: Figure S1), the cells were collected at points throughout lag phase, logarithmic growth phase, and stationary phase after a cultivation of 2, 3, 4, 5 and 6 days by centrifugation at 1136×*g* for 10 min at 4 °C (HC-3018R, Anhui USTC Zonkia Scientific Instruments Co., Ltd., Anhui, China). Next, the collected cells were washed twice with sterile water and immediately stored in liquid nitrogen until further analysis. The resting cells were collected using the method reported by Zhang et al. (2013a) as follows: after cultivation for 4 days, the rinsed cells were resuspended in 0.2 mol/L pH 7.0 phosphate buffer containing 0.1 g/L MgSO_4_, 0.2 g/L CaSO_4_ and 4 mmol/L phenylalanine for 21 h. After that, cells were washed twice with sterile water and stored for further analysis.

### Total RNA extraction and cDNA synthesis

Extraction of the total RNA from the cells was performed using a Spin Column Fungal total RNA Purification Kit (Sangon Biotech (Shanghai) Co., Ltd., China). Quality and quantity of the RNA extraction were analyzed using a NanoDrop 2000 Spectrophotometer (Thermo Scientific, Waltham, Massachusetts, USA) gel electrophoresis, and Agilent 2100 Bioanalyzer (Agilent Technologies, PaloAlto, California, USA). First-strand cDNA was synthesized using a PrimeScript™ RT reagent Kit with gDNA Eraser (Perfect Real Time) in strict accordance with the manufacturer’s operation manual (TAKARA Biotechnology (Dalian) CO., LTD., China). The cDNA products were diluted fivefold and stored at −20 °C before further analysis.

### Primer design of reference genes and qRT-PCR amplification conditions

The nine candidate genes (Table 1), *ACTB*, *EF1*, *EF2*, *RPS5*, *RPS24*, *TUBA*, *UBC*, *UFD* and *18S* were selected based on the transcriptome database of *Alternaria* sp. MG1 (available through NCBI, SRA study accession number SRP060338) (Che et al. 2016b). The internal reference genes had highly similar sequences with reported genes from previous studies (DiGuistini et al. 2011; Farajalla and Gulick 2007; Goodwin et al. 2011; Skora et al. 2015). The primer pairs of candidate genes were designed using the software Primer Premier (version 5.00) (http://www.premierbiosoft.com/primerdesign/index.html) with an amplicon length ranging from 100 to 300 bp.

**Table 1:** Relation of primers for the candidate genes to internal control

Internal gene	Gene name	Primer sequence (5′–3′) Forward/reverse	Amplicon length (bp)	Amplification efficiency (%)	Regression coefficient (*R* ^2^)	Accession number at GenBank
*ACTB*	β-Actin	CAAGACGGAAGGCTGGAA/ CACTGCCGAGCGAGAAAT	195	100.4	0.997	GEMY01018051
*EF1*	Elongation factor 1	CACTGGTTTTGCCTTTTCCT/ TGTGGGCACCGTCAAAGT	186	127.3	0.995	GEMY01015044
*EF2*	Elongation factor 2	ATAACAGCCTGGAAGATGC/ CTTTCACCATCCGTCAGTT	207	98.3	0.996	GEMY01001243
*RPS5*	Ribosomal protein S5	ACACCCATACAAAGAACG/ CCGAGTGCCTTGCTGA	131	104.1	0.985	GEMY01011888
*RPS24*	Ribosomal protein S24	CCGTCTTGTCGTTCCC/ CGATTGGCGGTTTCTC	133	104.8	0997	GEMY01015522
*TUBA*	α-Tubulin	CAAGCGAGTCAGAAGC/ GGTATGTTGGTGAGGGTAT	101	106.9	0.984	GEMY01012167
*UBC*	Ubiquitin-conjugating enzyme	GGCTCAAGAAACAGGAA/ AGATTTACCACCCGAAC	123	100.4	0.984	GEMY01016137
*UFD*	Ubiquitin fusion degradation protein	TCCTCCTTGCCCTTGA/ CGAATCCGCCTCCTAC	108	123.6	0.996	GEMY01001986
*18S*	18S ribosomal RNA	TCTTGTTTCCTTGGTGGGT/ GCATTTCGCTGCGTTCT	144	106.2	0.980	JN102357.1

The real-time quantitative PCR amplification and analysis were performed in the Bio-Rad iQ™5 Multicolor Real-Time PCR Detection System (Bio-Rad Laboratories, Inc., Hercules, California, USA) with the iQ™5 Optical system Software Version 2.1. (http://www.bio-rad.com/zh-cn/sku/1709753-iq5-optical-system-software?parentCategoryGUID=2). A total reaction system of 25 μL contained SYBR *Premix Ex Taq*II (Tli RNase Plus) (2× Conc.), 12.5 μL; PCR primer mix (10 μM), 2 μL; cDNA template, 1 μL; and DNase-free water, 9.5 μL. The qRT-PCR amplification program was 95 °C for 5 min, followed by 40 cycles of 94 °C for 30 s, the ideal annealing temperature for each primer pair for 30 s, and 72 °C for 1 min, and then 72 °C for 10 min. All reactions were conducted in triplicate and melting curve analysis was performed. The correlation coefficients (*R* ^2^) and slope values of the standard curve and efficiency (*E*) were calculated using the iQ 5 Optical system Software Version 2.1.

To confirm the accuracy of the amplified products, all the PCR products were analyzed by agarose gel electrophoresis using 2% agarose gels in Tris-borate-EDTA (TBE) buffer stained with ethidium bromide.

### Determination of reference gene expression stability using data analysis software

The transcript abundance of the reference genes was determined by the Ct value. The expression stability of these candidate reference genes were evaluated using the four methods described below. The methods are Excel-based tool—Bestkeeper software (version 1) (http://bioinformatics.gene-quantification.info/bestkeeper.html)using pair-wise correlations (Pfaffl et al. 2004), Genorm software (version 3.4) (https://genorm.cmgg.be/) (Vandesompele et al. 2002), NormFinder software (version 0.953) (http://moma.dk/normfinder-software/) (Andersen et al. 2004), and the comparative ∆Ct method (Pfaffl 2001; Silver et al. 2006). To comprehensively analyze the stability of these candidate genes, the web tool RefFinfer (http://fulxie.0fees.us/?type=reference) (Xie et al. 2012) was used to compare and rank the outcomes of the results using the different analysis programs.

## Results

### RNA purity and concentration

The mean values of quantity and quality of the RNA samples are shown in Table 2. The concentrations of RNA samples ranged from 324.00 to 1329.56 ng/μL. The mean values of 260/280 were close to 2.00.

**Table 2:** The quantity and quality of RNA samples isolated from *Alternaria* sp. MG1 during different growth stages

Sample	2 days	3 days	4 days	5 days	6 days	Resting cell
260/280	2.18	2.16	2.13	1.97	2.09	2.00
260/230	1.61	1.57	1.65	1.41	1.60	1.52
Conc. (ng/μL)	1329.56	762.96	563.76	324.00	336.84	594.04

### Verification of primer specificity of selected reference genes

**Fig. 1:**
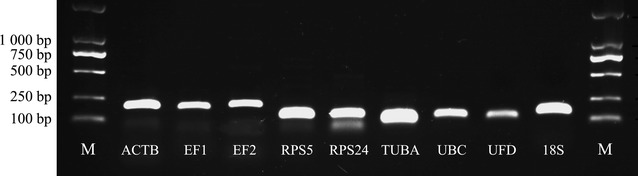
Amplification of the candidate reference genes from cDNA templates. Agarose gel electrophoresis shows amplification of a specific PCR product of the expected size for each gene

A total of nine candidate genes (Table 1) were selected for this study by referring to previous studies (DiGuistini et al. 2011; Farajalla and Gulick 2007; Goodwin et al. 2011; Skora et al. 2015) and the *Alternaria* sp. MG1 transcriptome database. Agarose gel electrophoresis for preliminary PCR and melting curve analysis was performed and the results are shown in Fig. 1 and Additional file 1: Figure S1, respectively. We observed that the lengths of amplified fragment were consistent with the expected size, and no primer dimers were detected except for candidate gene *RPS24*. These results indicated the primers were specific and suitable for reference gene validation.

PCR efficiency analysis was performed to validate the optimal of the reference gene. The regression coefficient (*R* ^2^ value) and PCR amplification efficiency were calculated by a standard curve generated using tenfold serial dilutions of pooled cDNA. The PCR amplification efficiency of these candidate genes ranged from 98.3 to 127.3%, and the regression coefficient (*R* ^2^ value) of the standard curve ranged from 0.980 to 0.997, well within the acceptable range of qRT-PCR (Table 1).

### Expression profiling of the candidate reference genes

Six samples were chosen for each candidate reference gene in this study.

**Fig. 2:**
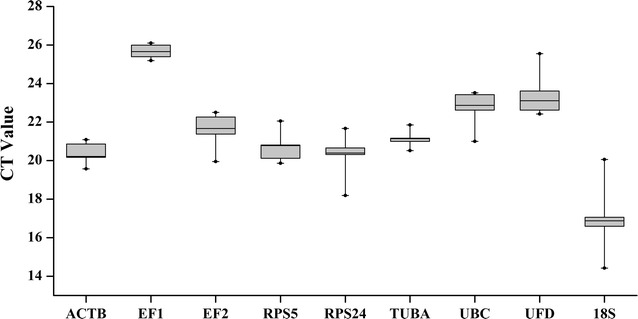
Expression profiling of nine reference genes in the experimental set of *Alternaria* sp. MG1. *Box* represents 25/75 percentiles, whisker cap represents 10/90, the *line* in the *box* shows the median, and the *dot* indicates outlier of min and max value

The average expression of the candidate genes during different growth stages was investigated by comparison of Ct values and the results are shown as a box-plot (Fig. 2). In the figure, the interquartile values are shown in boxes. The median expression level and the total expression level are shown as a line and whisker, respectively. The expression level of the nine reference genes with Ct value ranged from 14.22 to 25.66. Lower Ct values indicate higher expression level and vice versa. The *18S* gene showed the highest expression level, and the *EF1* was the lowest. There was little difference among the other candidate reference genes with Ct values between 20.24 and 23.38. Genes that showed different ranges of expression (Ct_max−_Ct_min_) were *ACTB* (1.52), *EF1* (0.91), *EF2* (2.55), *RPS5* (2.19), *RPS24* (3.48), *TUBA* (1.33), *UBC* (2.52), *UFD* (3.13), and *18S* (5.64).

### Stability evaluation of candidate genes using different analysis programs

To validate the stability of these nine candidate genes, we used four evaluation methods. The Bestkeeper software was employed to validate and rank the stability evaluation of these candidate genes based on the standard deviation (SD) of Ct values and the coefficient of variance (CV) expressed as a percentage of the Ct values. In this approach, the most stable reference gene was identified by the comparison of SD value and CV value of these selected genes. The lowest SD and CV values represent the genes with a highest stability, and vice versa. Here, the descriptive statistics of these nine candidate genes were calculated based on the Ct values, and the statistical outcome is listed in Table 3.

**Table 3:** CT data of reference genes calculated using Bestkeeper

Genes	*ACTB*	*EF1*	*EF2*	*RPS5*	*RPS24*	*TUBA*	*UBC*	*UFD*	*18S*
Number of sample	6	6	6	6	6	6	6	6	6
GM (CT)	20.35	25.66	21.56	20.72	20.24	21.13	22.7	23.38	16.9
AM (CT)	20.35	25.66	21.57	20.73	20.27	21.13	22.72	23.4	16.98
Min (CT)	19.57	25.19	19.95	19.86	18.19	20.52	21	22.42	14.42
Max (CT)	21.09	26.1	22.5	22.05	21.67	21.85	23.52	25.55	20.06
SD (±CT)	0.41	0.25	0.61	0.49	0.69	0.25	0.6	0.78	1.05
CV (% CT)	2.04	0.99	2.8	2.38	3.42	1.2	2.66	3.35	6.2
Min (x-fold)	−1.71	−1.39	−3.04	−1.82	−4.15	−1.53	−3.25	−1.95	−5.59
Max (x-fold)	1.67	1.36	1.92	2.51	2.69	1.65	1.76	4.5	8.93
SD (x-fold)	1.33	1.19	1.52	1.41	1.62	1.19	1.52	1.72	2.08

The geometric mean (GM), arithmetic mean (AM), extremum (min and max) value, standard deviation (SD), and coefficient of variation (CV), were calculated. Sorted by SD values, the tested genes were in the order of *EF1* < *TUBA* < *ACTB* < *RPS5* < *UBC* < *EF2* < *RPS24* < *UFD* < *18S*. The overall variation of *EF1* and *TUBA* were lowest with a SD value of 0.25. The CV values of *EF1* and *TUBA* were lower than the others, 0.99 and 1.2%, respectively. Interrelated analysis provided by BestKeeper concluded that the most stable reference gene was *EF1*, and *TUBA* was the second most stable.

**Fig. 3:**
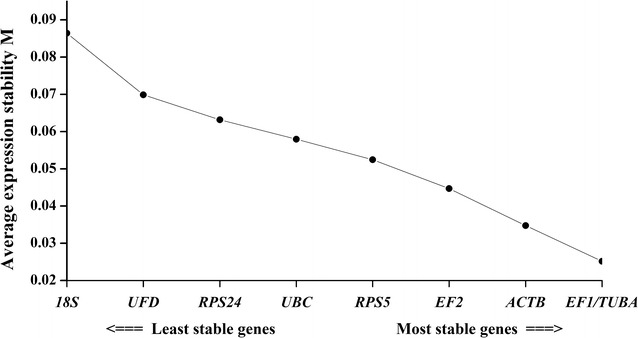
Average expression stability values (M) of the nine candidate reference genes as calculated by geNorm

**Fig. 4:**
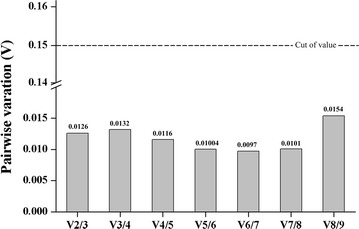
Pairwise variation (V) calculated by geNorm to determine the optimal number of reference genes

Another program, geNorm (Vandesompele et al. 2002), was used to calculate the average expression stability of M value and analyze the stability of the candidate reference genes. The calculated M values of the nine candidate reference genes are plotted in Fig. 3. The most stable expression genes had the lowest M value, and vice versa. Previous studies (Vandesompele et al. 2002; Wu et al. 2014), suggested selection of stable reference genes with M values below the threshold of 1.5. As shown in Fig. 3, all the M values of the tested genes were less than 1.5. In the growth stage and the resting cells, the *EF1* and *TUBA* showed the highest expression stability with the lowest M values (0.025). Sometimes, normalization with a single reference may produce significant errors and the more than one reference genes may be needed in some experiments. However, some researchers (Kong et al. 2015; Li et al. 2015) have indicated that multiple reference genes could increase instability and experimental complexity. Thus, pairwise variation (Vn/Vn + 1) was selected for assessing the optimal number of reference genes. The pairwise variation V value was calculated using geNorm, and a threshold V value of 0.15 was recommended to identify the number of the additional reference genes (Vandesompele et al. 2002). In Fig. 4, all the V values were below the cutoff value of 0.15. Pairwise variation analysis showed that the V2/3 value was 0.0126, which indicated two reference genes was sufficient for gene expression normalization and the two stability reference genes selected were *EF1* and *TUBA*.

**Fig. 5:**
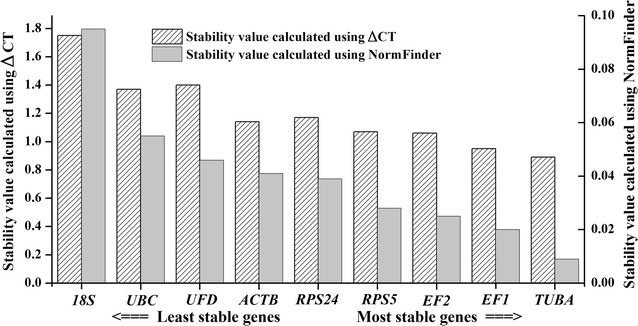
Stability values of the nine candidate reference genes as calculated using ΔCt and NormFinder

In an alternate approach, we used the Normfinder software program to evaluate these candidate reference genes. As a model-based variance estimation approach, Normfinder is used to calculate stability values and evaluate the expression stabilities of the tested genes (Andersen et al. 2004; Maroufi et al. 2010). A lower average expression stability indicated genes that were stably expressed gene. In Fig. 5, the stability value ranking of these candidate reference genes was slightly different from that calculated by geNorm software. However, the most stable reference gene was the same (*TUBA*), followed by *EF1*, *EF2*, *RPS5*, *RPS24*, *ACTB*, *UFD*, *UBC* and *18S*. The comparative ΔCt method was used to assess gene expression stability. The stability results were the same as those calculated using Normfinder software, and these results are shown together in Fig. 5. Again, the most stable reference gene was *TUBA*.

### Overall ranking order and selection of optimal reference genes

**Fig. 6:**
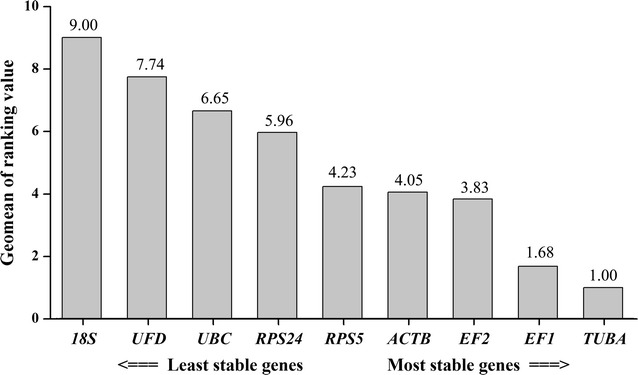
Ranking candidate reference genes estimated using RefFinder

In the separate assessments, the most stable reference gene was the same, but the other genes were ranked differently in the different analyses. Next, we used the web tool (RefFinder) to arrange the comprehensive results by integration of the results of the four assessments to compare these potential reference genes. An overview of the expression stability of the nine candidate genes from different growing stages and different treatment of *Alternaria* sp. MG1 are shown in Fig. 6. The ranking of these candidate reference genes (from most stable to least stable) were *TUBA*, *EF1*, *EF2*, *ACTB*, *RPS5*, *RPS24*, *UBC*, *UFD*, and *18S*.

## Discussion

As a bioactive polyphenol, resveratrol has a variety of functions, such as preventing or slowing the occurrence of cancer, acting as a powerful antioxidant, and extending life span. Pharmaceutical production and functional food processing present a high demand of resveratrol. To date, resveratrol was provided by extraction from plant materials. This method of production is highly limited by plant growth times and low yields. Many resveratrol-producing *Escherichia coli* or yeasts have been constructed by genetic modification (Conrado et al. 2012; Krivoruchko and Nielsen 2015). However, these processes have low yield and have stability issues during production due to the use of the plant-derived genes. The problem of low yield results from a complicated metabolic pathway and a rate-limited enzyme. In a previous study, resveratrol production was increased slightly by adding substrates or production in *Alternaria* sp. MG1 resting cell culture (Zhang et al. 2013b). Thus, understanding the expression of the genes involved in resveratrol biosynthesis pathway is the key problem to be solved. As a newly characterized biological resource, *Alternaria* sp. MG1 is able to produce resveratrol without limitation from plant resources (Che et al. 2016a; Shi et al. 2012). Study of this fungus may allow insight into the necessary pathways allowing engineering for increased production.

With several advantages, including the ability to quantify, reproducibility, sensitivity and accuracy, qRT-PCR is a preferred method to use for quantifying the gene expression, and assessing mRNA levels among different samples. Validation of appropriate reference genes is a prerequisite for accurate analysis of gene expression using qRT-PCR (Li et al. 2016). In the past research, the most traditional reference genes used in qRT-PCR assay were genes such as *ACT*, *TUB*, and *18S*. In the past, many studies showed that the expression of these traditional reference genes was not always stable under all conditions (Dankai et al. 2015; Zhou et al. 2012). The identification of the most stable reference genes in *Alternaria* sp. MG1 has not been achieved until now, although some traditional reference genes have been used for qRT-PCR data normalization in some other *Alternaria* sp. (Dankai et al. 2015; Sihto et al. 2014).

Several methods have been recently used to determine the stability of gene expression and to validate the best reference genes (Tong et al. 2009). However, there is no consensus on the ideal approach that should be used to examine the stability of reference gene expression. The pairwise comparison strategy, accessible through the geNorm software, is a very popular option to verify the expression stability of candidate genes (Yu et al. 2016). However, co-regulated genes may confound the geNorm software and his would lead to an erroneous choice of optimum normalizer pair (Andersen et al. 2004). To investigate whether the potential co-regulated genes affected the outcome of the results, researchers removed one of the co-regulated genes from analysis and reported that co-regulation did not influence the ranking of reference genes by stability (Tong et al. 2009); we similarly found no effect on the ranking by the inclusion of co-regulated genes (Additional file 1: Figure S2).Additionally, the reference genes that belonged to the same functional class were not top-ranked and did not occupy closed positions by geNorm software in previous studies (Exposito-Rodriguez et al. 2008). Similarly, the co-regulated genes were not top-ranked and did not occupy closed positions in this research. As a result, use of these two pairs of co-regulated genes did not affect the final ranking of the reference genes by using geNorm software. Other methods such as NormFinder and BestKeeper, were reported to be less sensitive to co-regulation, and might serve as appropriate statistical applets to further assess the stability for reference gene expression (Huang et al. 2014). In an effort to ensure the accuracy of the reference gene stability ranking and minimize bias introduced by the validation approach, four different statistical approaches, ∆Ct, geNorm, NormFinder, and BestKeeper, were used to identify the suitable reference genes for accurate normalization in this study. The overall ranking of these four approaches was integrated using a web-based comprehensive tool (RefFinder) developed to identify the most reliable reference genes by integrating these four evaluation methods (Xie et al. 2012).

Nine traditional reference genes (*TUBA*, *UFD*, *RPS24*, *RPS5*, *UBC*, *EF1*, *EF2*, *ACTB* and *18S*) were selected as candidate reference genes, and these genes were tested during different growth periods and during an optimized condition for resveratrol production. Different statistical algorithms and analytical methods gave different validation results. The overall ranking of these results was integrated using the RefFinder system. The comprehensive results of this research demonstrated that *TUBA* and *EF1* were the most stable reference genes, *18S* was the least stable gene, and the other candidate reference genes were intermediate among all six sets of experiments. Interestingly, *TUBA* was shown to be a reliable reference gene for *Penicillium expansum* (De Clercq et al. 2016) and *Valsa mali* var. *mali* (*Vmm*) (Yin et al. 2013), and *EF1* was found to be suitable for *Clonostachys rosea* (Sun et al. 2015) and *Tuber melanosporum* (Cesare et al. 2015). However, *TUBA* and *EF1* were unstable and unsuitable for use as reference genes in *Blumeria graminis* (Pennington et al. 2016), *C. rosea* (Sun et al. 2015), and *Pandora neoaphidis* (Chen et al. 2016). Other candidate reference genes showed different stability in different fungi. For example, the most stable reference gene for *Talaromyces marneffei* was *GAPDH*, followed by *TUBA*, and *ACTB* (Dankai et al. 2015). *ACTB* was identified as the reliable reference gene in *Penicillium echinulatum* (Zampieri et al. 2014).

Additionally, normalization with the combination of more genes resulted in improved accuracy. Previous research indicated the application of individual or combinations of 2, 3, and 4 reference genes would result in different levels of abundance, but qualitatively similar patterns (Hu et al. 2009). How many reference genes should be used is dependent on the purpose of research. One reference gene would be enough to show a rough expression mode of genes, if the reference gene was identified as a stable expressed gene (Cho et al. 2014). Nevertheless, if the research purpose is to compare gene expression levels among different samples or to get an accurate expression level, the more reference genes used, the more accurate the result is. However, other researches have reported that multiple reference genes could increase instability and experimental complexity (Kong et al. 2015; Li et al. 2015). Thus, pairwise variation (Vn/Vn + 1) was selected to assess the optimal number of reference genes. The pairwise variation V value was calculated using geNorm, and a threshold V value of 0.15 was recommended to identify the number of the additional reference genes (Sumby et al. 2012; Vandesompele et al. 2002). In this study, pairwise variation analysis (Fig. 4) showed that the V2/3 value was 0.0126, which indicated that two reference genes was sufficient for gene expression normalization and the two stability reference genes selected were *EF1* and *TUBA*.

In conclusion, the results obtained here and in previous studies indicate that validation of reference genes is crucial for accurate normalization of gene expression measurements under different experimental conditions. The reference genes verified in this study will be useful for future research to explore gene expression, molecular mechanisms, and improvement of secondary metabolite yields in *Alternaria* sp. MG1. To our knowledge, this was the first validation of reliable reference genes in *Alternaria*. The results of this study provide useful guidelines for the selection of reference genes in other *Alternaria* species.

## Additional file

**Additional file 1.** **Figure S1.** The growth curve of *Alternaria* sp. MG1 in liquid potato-dextrose broth (PDB) at 28 °C and 120 rpm. **Figure S2.** Average expression stability values (M) of reference genes removed one of the coregulated genes as calculated by geNorm.
