# Supplementary material for: Validation of reference genes for normalization of gene expression by qRT-PCR in a resveratrol-producing entophytic fungus (Alternaria sp. MG1)
Source: AMB Express. 2016 Nov 8;6:106. doi: 10.1186/s13568-016-0283-z (PMC5101243; doi:10.1186/s13568-016-0283-z)
Supplement: Supplementary file 1 — Additional file 1. Figure S1. The growth curve of Alternaria sp. MG1 in liquid potato-dextrose broth (PDB) at 28 °C and 120 rpm. Figure S2. Average expression stability values (M) of reference genes removed one of the coregulated genes as calculated by geNorm. [file 13568_2016_283_MOESM1_ESM.pdf]

AMB Express

Validation of reference genes for normalization of gene expression by qRT-PCR in a resveratrol-producing entophytic fungus (*Alternaria* sp. MG1)

Jin-xin Che [1], Jun-ling Shi[2]*, Yao Lu[2], Yan-lin Liu[1†]

[1] College of Food Science and Engineering, Northwest A & F University, 28 Xinong Road, Yangling, Shaanxi 712100, China

[2] Key Laboratory for Space Bioscience and Biotechnology, School of Life Sciences, Northwestern Polytechnical University, 127 YouyiXilu, Xi'an, Shaanxi Province 710072, China

* Corresponding author: Junling Shi, Tel.: +86 29 88460541; fax: +86 29 88460541; E-mail: sjlshi2004@nwpu.edu.cn

† Co-corresponding author: Yan-lin Liu, Tel.: +86 29 87091994, fax: +86 29 87091994, E-mail: yanlinliu@aliyun.com

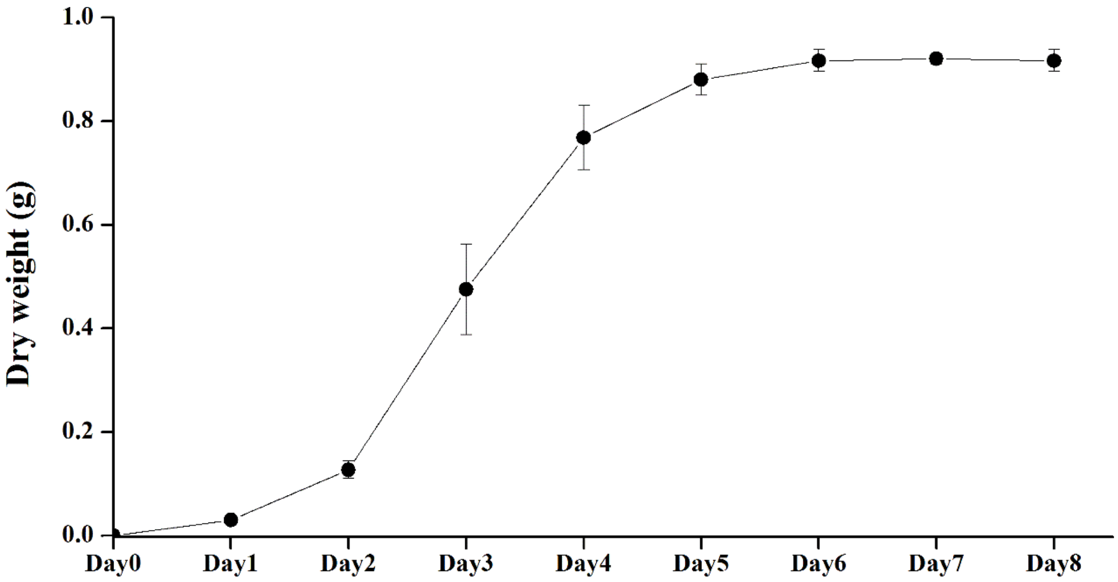

Fig. S1 The growth curve of *Alternaria* sp. MG1

in liquid potato-dextrose broth (PDB) at 28°C and 120 rpm

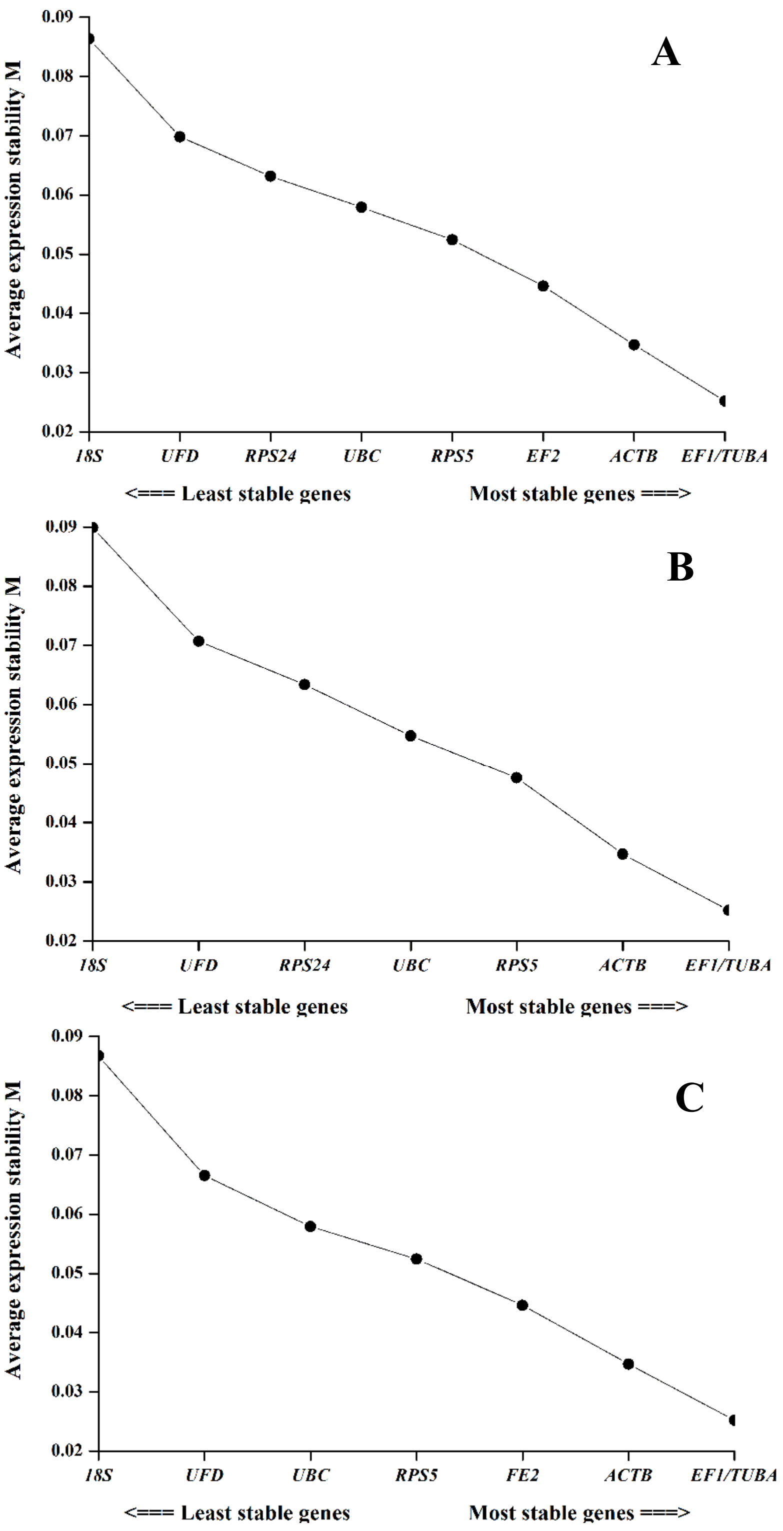

Fig. S2 Average expression stability values (M) of reference genes removed one of the co-regulated genes as calculated by geNorm.
